# Assessment of Dry Epidermal Electrodes for Long-Term Electromyography Measurements

**DOI:** 10.3390/s18041269

**Published:** 2018-04-20

**Authors:** Momona Yamagami, Keshia M. Peters, Ivana Milovanovic, Irene Kuang, Zeyu Yang, Nanshu Lu, Katherine M. Steele

**Affiliations:** 1 Department of Electrical Engineering, University of Washington, Seattle, WA 98195, USA; my13@uw.edu; 2 Department of Mechanical Engineering, University of Washington, Seattle, WA 98195, USA; rumbek@uw.edu (K.M.P.); ivamil@uw.edu (I.M.); 3 Department of Electrical Engineering and Computer Science, Massachusetts Institute of Technology, Cambridge, MA 02139, USA; ikuang@mit.edu; 4 Chengdu Rotex Technology Company Ltd., Chengdu 610041, China; zeyu.yang@rotextech.com; 5 Department of Biomedical Engineering, Aerospace Engineering and Engineering Mechanics, University of Texas, Austin, TX 78712, USA; lunanshu@gmail.com

**Keywords:** neurological injury, stroke rehabilitation, impedance measurements, Jebsen Taylor Hand Function Test, Box & Block, signal-to-noise ratio

## Abstract

Commercially available electrodes can only provide quality surface electromyography (sEMG) measurements for a limited duration due to user discomfort and signal degradation, but in many applications, collecting sEMG data for a full day or longer is desirable to enhance clinical care. Few studies for long-term sEMG have assessed signal quality of electrodes using clinically relevant tests. The goal of this research was to evaluate flexible, gold-based epidermal sensor system (ESS) electrodes for long-term sEMG recordings. We collected sEMG and impedance data from eight subjects from ESS and standard clinical electrodes on upper extremity muscles during maximum voluntary isometric contraction tests, dynamic range of motion tests, the Jebsen Taylor Hand Function Test, and the Box & Block Test. Four additional subjects were recruited to test the stability of ESS signals over four days. Signals from the ESS and traditional electrodes were strongly correlated across tasks. Measures of signal quality, such as signal-to-noise ratio and signal-to-motion ratio, were also similar for both electrodes. Over the four-day trial, no significant decrease in signal quality was observed in the ESS electrodes, suggesting that thin, flexible electrodes may provide a robust tool that does not inhibit movement or irritate the skin for long-term measurements of muscle activity in rehabilitation and other applications.

## 1. Introduction

Surface electromyography (sEMG) is a method that uses electrodes placed on the surface of the skin to record muscle activity [1,2]. These signals are valuable in a variety of applications, including diagnosis of neuromuscular diseases and evaluations of muscle function [3,4,5]. Many fields including athletics, rehabilitation, and surgical monitoring routinely use sEMG data to monitor muscle activity and guide interventions. While sEMG is commonly used to evaluate the timing and magnitude of muscle activation, sEMG can typically only be captured in lab or clinic environments and there are few methods available to collect high-quality sEMG signals for longer than a few hours [6,7,8,9]. 

Currently, wet gel electrodes and dry metal electrodes are used for short-term sEMG data collection. Standard silver-silver/chloride gel electrodes provide quality sEMG measurements, but are limited to short-term use due to skin irritation and signal degradation as the gel dries out over time [3,7]. Conventional dry electrodes can also only be used for short periods of time due to high device costs, subject discomfort, and concerns about skin health [10]. Such systems must be placed close to a base station or wired to a data acquisition system to transmit sEMG data, preventing patients from being measured remotely in ambulatory conditions. Inflexible metal electrodes also suffer from high motion artifacts due to an inability to conform to the contours of the skin [10]. Nevertheless, dry electrodes provide a foundation for improvement and development of long-term sEMG solutions.

In recent years, a variety of dry electrodes, such as the Myo and the BioStamp, have been developed and commercialized to provide affordable, ambulatory alternatives to conventional electrodes. The Myo (Thalmic Labs, Inc., Kitchener, ON, Canada) is an armband with eight rigid electrodes that can be used on the forearm for gesture recognition, enabling device-free interaction with technology [11,12], playing instruments [13], sign language interpretation [14,15], and other applications. The BioStamp (MC10, Lexington, MA, USA) is a silicone-based flexible system for sensing muscle activity and movement. It has been successfully used to monitor people with mobility impairments such as Parkinson Disease and Huntington Disease [16,17]. However, battery and memory management limit use of these devices to a few hours and electrode placements have not been optimized for targeted muscle monitoring, especially for smaller muscles.

To help address these concerns, a variety of dry electrodes have been developed with flexible polymers and textiles that allow the electrode to adhere and move with the skin. For electrodes with a polymer adhesive, thin sheets of metal [10,18,19,20,21,22,23], carbon ink [24] and powders [25], as well as atomically thin sheets such as graphene [26] have been used as a conductive material. Textile-based electrodes with silver-plated conductive thread have also been reported [27]. Various measures have been used to characterize the quality of these electrodes. The most common are electrode-skin contact impedance and signal-to-noise ratio (SNR). While lower impedance is desirable for better conduction [28], dry electrodes tend to have a much higher impedance than wet electrodes, on the order of high kΩ-high MΩ [7,10,22,29], whereas wet electrodes have an impedance on the order of low kΩ-low MΩ [24,30]. For long-term sEMG data measurements, constant impedance over time is desirable to ensure detected changes reflect true changes in muscle activity and not changes in the sensing environment. One study using a novel polymeric dry electrode over seven days demonstrated little to no change in contact impedance [10]. SNR is also often used as a measure of signal quality and can range from ~10 dB [16] to 50 dB under ideal, simulated conditions [31]. Another study testing a graphene-based electrocardiography electrode used SNR in a week-long test to validate high signal quality [32].

While measurements of skin impedance and SNR provide useful metrics in the evaluation and design of novel electrodes for long-term sEMG measurements, there are limitations in relying solely on these metrics for developing electrodes suitable for rehabilitation or other applications. There is limited prior research analyzing sEMG quality across multiple muscle groups in settings relevant to clinical or home environments. For example, in the context of rehabilitation, an understanding of sEMG electrode performance in common upper extremity clinical tests is needed. Prior research primarily focused on large muscles (e.g., biceps or quadriceps [3,29,33]), which are easier to monitor with sEMG electrodes, and evaluated tasks with large muscle activity, such as maximum voluntary isometric contractions (MVICs). The performance of sEMG electrodes for smaller muscles (e.g., specific forearm muscles) during tasks that are more representative of clinical tests or tasks of daily living remain unclear [34,35]. To enable long-term sEMG monitoring in the clinic, it is crucial to test signal quality across muscle groups and during less demanding and clinically relevant tests.

**Figure 1:**
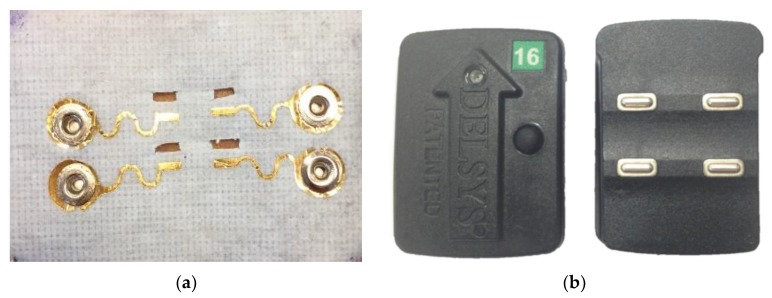
(**a**) ESS electrode with gold filament on physiological tape. The electrode was designed to allow for simultaneous measurements from both the Delsys and ESS systems. The electrodes are designed to have a similar contact area and configuration as the Delsys, at 5 mm in length and 1 mm in width, at a distance of 7.5 mm. (**b**) The Delsys Trigno Wireless System electrode.

In this work, we present the evaluation of the epidermal sensor system (ESS)—a low cost, thin, and flexible dry electrode that can be used with existing data acquisition systems for sEMG measurements (Figure 1) [23]. This system uses clinical-grade tape to support skin health during long-term wear while also providing a flexible substrate to maintain skin contact during dynamic activities. The flexible, gold-based electrodes are comprised of a metal-coated polymer on medical grade tape, which can be in any arrangement or layout desired. We analyzed the signal quality of the ESS compared to traditional dry electrodes that are currently used in many clinical and research applications (e.g., Delsys Trigno System, Boston, MA, USA), during both MVICs and standard clinical tests. The Delsys sensor is a widely-used and commercially-available reusable electrode comprised of 99.9% silver, with contact dimensions 5 × 1 mm. At full charge, these sensors can be operational for up to eight hours. We designed the ESS electrode layout to match the electrode size and distance of the Delsys sensors to limit variation in electrode size and spacing and provide a direct comparison between the systems. We used standard measures of signal quality including signal-to-noise ratio and signal-to-motion ratio. Measurements from ESS electrodes were compared against the Delsys electrodes over the course of six hours, and stability of ESS electrode signals was measured over four days.

## 2. Materials and Methods

### 2.1. Electrodes

The gold-based ESS electrodes (100-nm-thick gold on 13-µm-thick PET) were manufactured by the cost- and time-efficient “cut-and-paste” method [21]. This method allows us to use a minimal amount of gold to be used as the electrode, allowing each electrode to be low-cost compared to other dry electrodes, which use a significantly larger amount of metal. Current manufacturing costs of the ESS electrode are on the order of cents (<$0.10 US). For sEMG applications, we chose to support the electrodes with Kind Removal silicone tape (3M^TM^, St. Paul, MN, USA). This tape has a total thickness of 200 µm and Young’s modulus of 228 MPa [23], which means it is flexible without being stretchable. This tape was chosen because it is gentle on the skin and can be reattached more than 20 times, allowing a subject to remove the electrode as needed for swimming, showers, or other daily activities. To enable simultaneous measurements from the ESS and Delsys electrodes, four rectangular openings were cut in the tape for the four electrodes from the Delsys to protrude. The Delsys electrode was chosen as a comparison against the ESS electrode as the Delsys is a dry electrode system commonly used in clinical and research settings. The ESS electrodes were manufactured such that the corresponding rectangular electrodes for the Delsys unit would be 1 mm away from the ESS electrodes. The position and size of the electrode contacts were the same dimensions as the Delsys electrode contacts. Snap buttons were installed at the terminal pads for wire connection to a separate Delsys unit with snap-on leads. By using the Delsys system for data collection, this ensured that the data from the two electrodes were synchronized and underwent the same on-board signal processing. While simultaneous collection of sEMG data from both the Delsys and ESS electrodes prevent data collection from the same location, both electrodes were placed on the muscle belly to ensure that the electrodes were in an active muscle area and that the identical motion and activity was captured and compared for both electrodes. Except for the rectangular-shaped electrodes, all interconnects and snap buttons were encapsulated by insulating 3M^TM^ Nexcare liquid bandage spray such that the sEMG signals were only captured by the exposed rectangular electrodes. While non-polarizable materials such as silver are more commonly used for biopotential recordings due to its decreased likelihood of motion artifacts [36], the ESS electrodes are made out of corrosion-resistant polarizable gold which is favorable for extended continuous use [37].

### 2.2. sEMG Data Collection Over Six Hours

Eight subjects were recruited (four females, four males, all right-handed, age = 27.3 ± 3.7 years, height = 171 ± 11.3 cm, weight = 61.7 ± 15.3 kg) to collect sEMG and impedance recordings from four upper extremity muscles on the dominant arm—the extensor carpi radialis (ECR), flexor carpi ulnaris (FCU), biceps (BIC), and triceps (TRI). These muscles were selected to evaluate the signal quality of the ESS and Delsys electrodes for both large and small muscle groups. The electrodes were placed following Surface Electromyography for the Non-Invasive Assessment of Muscles (SENIAM) recommendations for BIC and TRI, and ECR and FCU placements were placed relative to bony landmarks and manual muscle testing. Excessive hair was removed, and the skin was wiped with an alcohol preparation pad and allowed to dry before attaching the ESS and Delsys electrodes. After confirming signal quality, the electrodes and wires were secured with a light wrap of Coban^TM^ (3M^TM^, St. Paul, MN, USA). The protocol was approved by the Institutional Review Board of the University of Washington Human Subjects Division under application number 47,743, and all subjects provided written consent.

To quantify and compare the signal quality of the ESS and Delsys electrodes, each subject performed muscle activation tests, including MVICs and dynamic range of motion tests, as well as functional tests including the Jebsen Taylor Hand Function Test [38] and Box & Block Test [39]. Muscle activation tests allowed us to evaluate the similarity of sEMG measurements and signal quality between the Delsys and ESS electrodes for a large range of sEMG values, while the functional tests helped determine the similarity of sEMG measurements and signal quality between electrodes for clinically-relevant tests that mimic movements performed in everyday life, which tend to have smaller sEMG amplitudes [40]. The Jebsen Taylor and the Box & Block Tests were chosen for their clinical relevance as both are standard unimanual tests commonly used clinically to assess individuals with neurologic injuries.

Each subject sequentially performed the following three activities:

1. Maximum voluntary isometric contractions (MVICs): While seated, the subject was asked to perform MVICs, contracting against a static support for each muscle. For the ECR and FCU, subjects were stabilized by the experimenter with their wrist at a 45° angle, and for the TRI and BIC, subjects were stabilized with their elbow at a 90° angle. Subjects were asked to rest for 5 s, contract against a support for 5 s, then rest for 5 s. Subjects were given loud verbal encouragement during contraction to ensure maximal effort.
2. Dynamic range of motion test (Dynamic): Subjects were asked to choose between a 2, 3, and 5 lb weight and perform a dynamic range of motion test with verbal and visual cues. For ECR and FCU, subjects held the weight 45° below a horizontal plane with their forearms parallel to the ground for 5 s, then over 2 s lifted the weight to a 45° angle above the horizontal plane and held the weight at this angle for 6 s, concluding by slowly releasing the weight over 2 s back to 45° below horizontal (ECR: forearm pronated; FCU: forearm supinated). For TRI, subjects held the weight in a lunge position with their upper arm parallel to the ground for 5 s, then over 2 s extended their forearm without moving their upper arm and held the extension for 6 s and concluded by lowering the weight over 2 s. For BIC, subjects were asked to stand and hold the weight with their arm relaxed for 5 s, then with their elbow tucked into their side and forearm supinated to lift the weight to a 90° angle (parallel to the ground) over two s, hold the weight for 6 s, and to conclude by slowly lowering the weight back down over 2 s.
3. Functional tests (Functional): For the Jebsen Taylor Hand Function Test [38], subjects were asked to complete the seven Jebsen Taylor tasks (writing, card flipping, small objects, simulated feeding, checkers, light objects, heavy objects) as quickly as possible with their dominant hand. The time taken to fully complete each task was recorded. For the Box & Block Test [39], subjects were asked to move wooden cubes (2.5 cm), one cube at a time from their dominant to non-dominant side over a 19 cm high partition as quickly as possible. Subjects were given a 15 s practice session, followed by a one-minute test moving the cubes. The number of cubes moved over the minute-long test was recorded. The sEMG signals taken from each trial were concatenated for analysis to provide an evaluation of the functional tests as a whole, instead of evaluating individual tasks (i.e., seven separate Jebsen Taylor tasks).

The MVIC test was conducted four times—once every two hours over six hours, while the dynamic test and functional tests were conducted twice, six hours apart to test for the stability of sEMG signals over time.

### 2.3. sEMG Analysis

The Delsys data acquisition system was used to collect sEMG data from both electrodes at 1926 Hz. The data was amplified by a factor of 909 and filtered on-board with a 20–450 Hz bandpass filter [41]. Sufficient line noise filtering was noted during data collection through the use of the real-time Signal Quality Tool included in the Delsys EMGworks**^®^** Acquisition software [42]. The data was subsequently processed in MATLAB (MathWorks, Inc., Natick, MA, USA). The measurements were high-pass filtered (40 Hz, 4th order Butterworth), rectified, and low-pass filtered (40 Hz, 4th order Butterworth) to obtain a linear envelope [43]. We chose a higher frequency for the low-pass filter because, although a lower cutoff frequency will provide higher correlations, it smooths out signal fluctuations during high-speed movements. We wanted to test the fidelity of the electrodes in challenging conditions, using a cutoff frequency that preserves the signal variations during quick movements while performing functional tests. The average amplitude of the linear envelope of the Delsys electrode was compared against the ESS electrode with the Wilcoxon signed rank test function signrank in MATLAB. Frequency domain measures including the power spectral density (PSD), signal-to-noise ratio (SNR), and signal-to-motion ratio (SMR) were calculated, as described below. Changes in Pearson’s correlations for the linear envelope, as well as SNR, and SMR were analyzed for stability across time and muscle type with a linear regression model. For the purposes of sEMG analyses, functional tests were concatenated across time for all tasks in the Jebsen Taylor and Box & Block trials.

Measures of signal quality were calculated as follows:

1. Pearson’s correlations: Correlations were calculated by taking the Pearson’s correlation for the linear envelope of the Delsys and ESS electrode. Correlation coefficients values between 0.7 and 1.0 were interpreted as a strong positive linear relationship. We defined a moderate correlation as values between 0.3 and 0.7, and low correlation as between 0.0 and 0.3.
2. Power spectral density (PSD): PSD was calculated using Welch’s PSD estimate with 50% overlap. Each segment was windowed with a Hamming window. The frequency content of sEMG data is generally between 5–500 Hz, with the most frequency power between 20–50 Hz [28,44].
3. Signal-to-noise ratio (SNR): Noise was assumed to be any signal present in the upper 20% of the frequency range (above 400 Hz) [31]. The total power across all frequencies was divided by the power of the frequency range above 400 Hz to obtain the SNR. An sEMG signal simulated under ideal conditions with minimum influence of motion and noise artifacts would have an SNR of at least 50 dB, while the sEMG signal influenced by noise would have an SNR of 15 dB or below [31]. A linear regression model was constructed to examine the effect of time, type of muscle, and test on SNR for the Delsys and ESS electrodes. For this calculation, line noise was assumed to be filtered out by the Delsys amplifier for both electrodes.
4. Signal-to-motion ratio (SMR): Two assumptions were made to calculate SMR: (1) that all motion noise is under 20 Hz, and (2) that the sEMG signal under 20 Hz is approximately linear [31]. As such, motion noise can be calculated by drawing a line drawn between 0 dB and the peak power between 50 Hz and 150 Hz, and calculating the power above the line from 0 to 20 Hz. The SMR is calculated by dividing the total power across all frequencies by the motion noise. A higher SMR indicates that the sEMG data are less affected by motion artifacts. A linear regression model was also constructed to examine the effect of time and type of muscle on SMR for the Delsys and ESS electrodes.
5. Linear regression models: MATLAB was used to run forward stepwise multiple linear regressions (stepwiselm) to determine which variables (time, muscles, tests, and electrodes) were associated with Pearson’s correlation, SNR, and SMR measurements. Fisher transformations were performed on the correlation measurements to obtain normal distributions for analysis. We used the MATLAB function stepwiselm to determine which predictors were important in predicting the output variables to find an optimal linear regression model. Next, the data was fit to the chosen linear regression model using the MATLAB function fitlm to evaluate the magnitude of significant changes with time, muscles, tests, and/or electrodes. While overfitting is a concern to a small sample size of eight participants, the robustops option was used when fitting the linear regression model to make the model less sensitive to outliers that may arise with a small sample. In addition, assumptions of normality were evaluated by plotting and assessing residuals.

### 2.4. Stability Testing of ESS Electrode over Seven Hours

Skin impedance of the ESS electrodes was measured over seven hours to evaluate the stability of electrode-skin contact. Impedance measurements were collected using the NanoZ (NeuraLynx, Bozeman, MT, USA). The impedance between the electrode and the skin was measured for each ESS electrode contact for all muscles. At each time point, impedances were collected at logarithmically equal frequency intervals between 4 Hz and 2 kHz for a total of 20 frequencies, with each measurement the result of averaging 40 measurements. The impedance was averaged over subjects and muscle groups for each frequency to obtain an impedance versus frequency graph at each time point. These frequencies were chosen for our measurements because the majority of sEMG power lies between 5–500 Hz and this range has been used in prior research [10,19,27]. Preliminary testing showed that impedance measurements change over hours, not minutes, so measurements were taken every 30 min for 2 h, then every hour for the next 5 h. Impedance measurements were collected only for the ESS electrodes, as the rigid, boxed design of the Delsys electrodes do not allow the user to directly evaluate contact impedance [41].

### 2.5. Stability Testing of ESS Electrode over Four Days

Four additional subjects were recruited (two females, two males, all right-handed, age = 25.3 ± 2.1, height = 170 ± 3.8 cm, weight = 66.2 ± 5.83 kg) to test the stability of the ESS electrode signals over four days. Long-term sEMG tests could only be performed for the ESS electrodes, since the form factor, cost, and battery life of the Delsys system does not enable long-term measurements. While a great clinical and research tool for use in the lab, the Delsys electrodes protrude from the skin and can be easily bumped or damaged if worn under clothing outside of the lab. The cost of the system also makes the risk for long-term wear outside of the lab infeasible for this research. Electrode placement was marked on the skin using a physiological marker, and subjects were instructed to keep the electrode on their skin at all times, except for when bathing or exercising. The skin was prepped with alcohol wipes only on the first day, and subsequent reapplications of the electrodes were done without skin preparation.

Impedance testing, as well as the sEMG tests (MVIC, Dynamic, and Functional) were conducted sequentially at approximately the same time each day. Average impedance measurements and linear envelopes for the sEMG tests were calculated, as well as the PSD, SNR, and SMR. Due to the small sample size of four participants, consistency of signal was evaluated by calculating intra-class correlation (ICC) to evaluate signal consistency while taking into account between-subject variability. ICC estimates were calculated using the MATLAB function ICC based on a mean-rating (*k* = 8), absolute-agreement, 2-way mixed-effects model for the impedance, and a single-rating, absolute-agreement, 2-way mixed-effects model for SNR. An ICC above 0.75 is indicative of good reliability, while an ICC between 0.5 and 0.75 indicate moderate reliability, and values below 0.5 indicate poor reliability [45]. The coefficient of variation (CV) was also calculated to determine the variation of the data relative to the mean.

## 3. Results

### 3.1. Comparison of ESS and Delsys Electrodes

**Figure 2:**
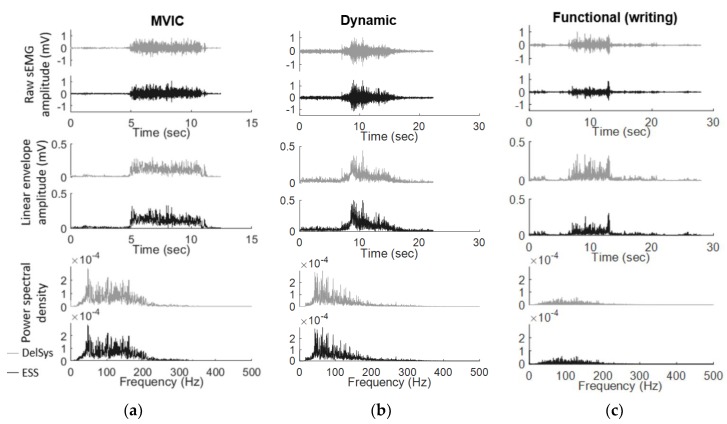
Sample sEMG signal from one subject’s FCU for (**a**) MVIC; (**b**) dynamic and (**c**) functional tests indicate that there were no significant differences between the Delsys (lighter grey) and ESS electrodes (darker grey) based on raw sEMG amplitude, linear envelope amplitude, or power spectral density.

Signals from the Delsys and ESS electrodes were highly correlated, demonstrating similar signal quality and recordings between the two electrodes. The raw sEMG data for the Delsys and ESS electrodes were similar in magnitude, as well as the timing of muscle activations in both the time and frequency domains (Figure 2). The Wilcoxon signed rank test demonstrated that the average linear envelope amplitude was greater for the ESS electrode compared to the Delsys (ESS average: 0.046 mV, Delsys average: 0.035 mV, *p* < 0.001).

**Figure 3:**
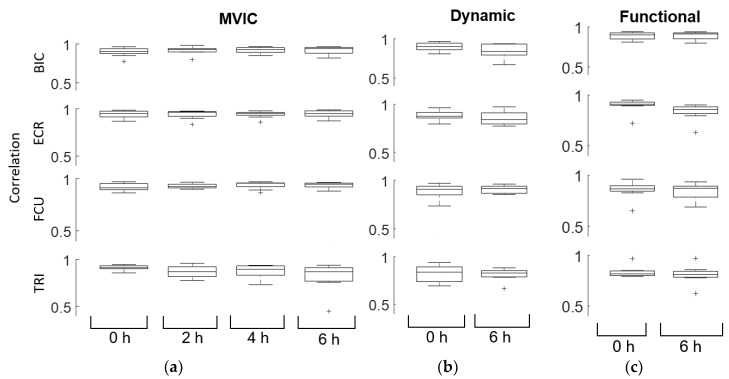
Linear envelope correlations for (**a**) MVIC, (**b**) dynamic, and (**c**) functional tests indicate high similarity between the Delsys and ESS electrodes. The rows illustrate the correlations for BIC, ECR, FCU, and TRI (top to bottom, respectively). Plus signs represent outliers. Correlation did not change significantly with time. The ECR had the highest correlation across time points and tests, followed by FCU, BIC, and TRI. The dynamic and functional tests performed slightly worse for ECR and TRI compared to MVIC (*p* < 0.05), and the functional tests performed slightly worse for FCU (*p* < 0.05) compared to MVIC.

Linear regression models of the correlation and SNR demonstrated similarity in sEMG measurements across all subjects, time points, and type of test conducted. The average correlation between the Delsys and the ESS electrodes was 0.89 ± 0.076 across all muscles, time points, and tests (Figure 3, Table 1). By fitting a linear model to the correlation, we determined that there were no changes in correlation over time, but that the correlations differed between muscles. ECR had the highest correlation across time points and tests, followed by FCU, BIC, and TRI. The dynamic and functional tests performed as well as MVIC for BIC, while the dynamic and functional test performed slightly worse for ECR and TRI (Dynamic and Functional ECR: *p* < 0.001, Dynamic TRI: *p* = 0.016, Functional TRI: *p* = 0.0085), and the functional test performed slightly worse for FCU (FCU: *p* = 0.001). However, all correlations were moderately high, above the 0.45 minimum measured correlation for TRI MVIC at 4 h, so while differences in signal correlation between the tests were statistically significant, the clinical difference was minimal. Although the functional tests were expected to have lower correlations than MVIC tests due to the lower overall sEMG amplitude generated from everyday activities, the ESS electrodes were highly correlated to the Delsys even during these tasks. Functional tests had an average correlation of 0.85 ± 0.08, while MVICs had an average correlation of 0.91 ± 0.07, and dynamic tests had an average correlation of 0.86 ± 0.07.

**Figure 4:**
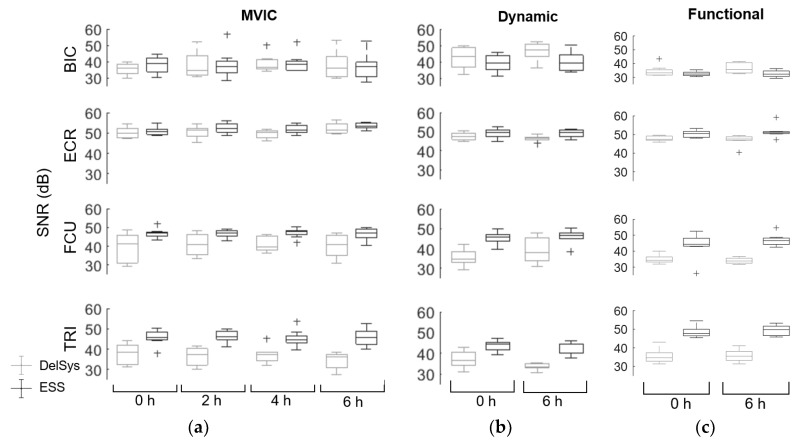
SNR for each muscle group across time points for (**a**) MVIC; (**b**) dynamic and (**c**) functional tests averaged across eight subjects for Delsys and ESS electrodes. Plus signs represent outliers. The BIC had a lower ESS SNR than the Delsys electrodes for the MVICs (*p* = 0.007), but the ESS SNR for FCU and TRI were higher for the MVICs than the Delsys electrodes (*p* < 0.001, *p* < 0.001). There was no significant difference in SNR for ECR.

Analysis of SNR for the Delsys (40.4 ± 7.0 dB) and ESS electrodes (45.1 ± 6.6 dB) indicated that SNR quality depended heavily on muscle type, with some muscles having a higher ESS SNR than the Delsys, and other muscles having a lower ESS SNR (Figure 4). The fitted linear regression model demonstrated that time did not affect SNR for either the Delsys or ESS electrodes. Thus, for both electrodes, SNR signal quality was consistent over the six hours measured. There was an interaction between the muscles and the electrodes, demonstrating that there were differences in SNR across muscles. For MVICs, the BIC had a lower ESS SNR than the Delsys (BIC: 34.3 dB, 37.2 dB, *p* = 0.0040), but FCU and TRI had a significantly higher ESS SNR than the Delsys (FCU: 47.6 dB, 38.6 dB, *p* < 0.001, TRI: 37.0 dB, 35.8, *p* < 0.001). There was no significant difference in the ECR SNR. The dynamic tests performed slightly better for some muscles and worse for others, while the functional SNR was slightly worse than the MVIC SNR for BIC, ECR, and FCU. The decrease was less than 5 dB for all tests and muscles, however, and the lowest SNR recorded was 27.4 dB for the Delsys electrodes, and 26.1 dB for the ESS electrodes, well above the 15 dB threshold for an EMG signal contaminated with noise [31]. The maximum SNR for the Delsys and ESS electrodes were 56.2 dB and 59.1 dB, respectively. While the MVIC tests still had the highest SNR values, the dynamic and functional tests still yielded signals sufficient for high-quality sEMG recordings.

The fitted linear regression model demonstrated that SMR was statistically higher for the Delsys electrodes than the ESS electrodes (*p* < 0.001) for all muscle types, with an average of 27.4 ± 12.9 dB and 14.4 ± 9.4 dB, respectively. There was no trend as to larger or smaller muscles having better SMR. This was not surprising given that the Delsys electrodes are self-contained, while the ESS electrodes required snap leads to connect to the Delsys monitoring system. Similar to SNR, there was no relationship between the time of the test and SMR, meaning that the SMR did not change over time, but there was a relationship between muscles and electrodes, and muscles and tests. The SMR for the dynamic and functional tests were equal to or worse than MVIC SMR depending on the muscle type (*p* < 0.01). This is consistent with the degree of movement in the respective tests, as the dynamic and functional tests had more overall movement of the upper extremity relative to MVIC. The minimum and maximum SMR for the Delsys electrodes was 0.9 and 60.7 dB respectively, and 0.7 and 39.0 for the ESS electrodes.

### 3.2. Signal Consistency of ESS Electrodes

**Figure 5:**
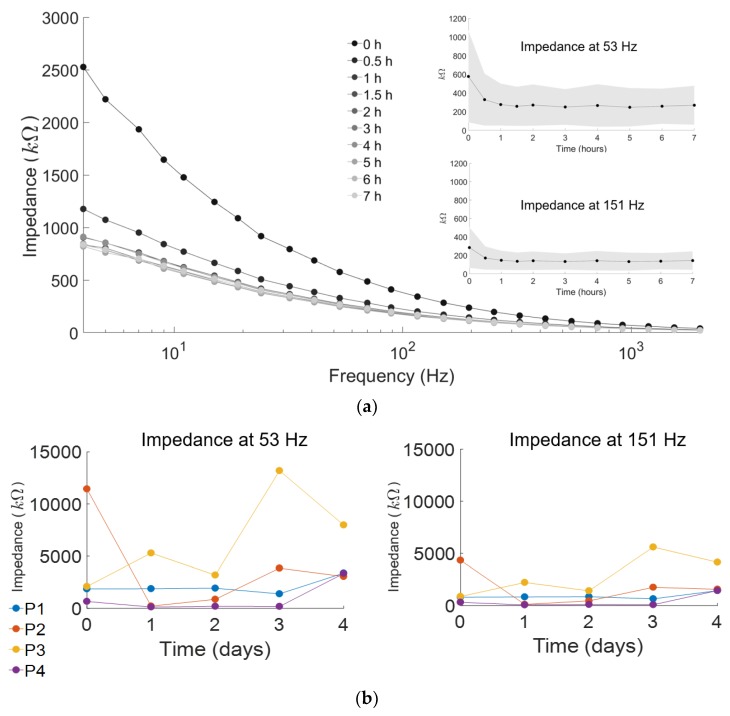
Impedance values measured across seven hours (*n* = 8 subjects) and across four days (*n* = 4 subjects). (**a**) Average impedance magnitudes were taken across each contact point of the electrode (*n* = 4) over all muscles. The impedance of ESS electrodes decreased for the first two hours before stabilizing for 2–7 h post-application. Impedance at 53 Hz and 151 Hz averaged across eight subjects demonstrate similar trends. (**b**) Impedance at 53 Hz and 151 Hz for the four subjects across four days demonstrates inter-subject variability in skin impedance over time. Each line represents an individual subject’s impedance.

Skin impedance recorded over the seven-hour and four-day trials indicated stable signals over time from the ESS electrodes (Figure 5). Impedance values decreased for the first two hours before stabilizing for the last five hours at an average of 252 kΩ at 53 Hz and 139 kΩ at 151 Hz (Figure 5a, insert). Of the 20 logarithmically equal intervals from 4 Hz–2000 Hz, these two frequencies were chosen to represent the varying stability of skin impedance since they fall within the 50–150 Hz window reported to encompass the majority of sEMG power [28,44].

Impedance recordings across four days showed no significant trend for each individual, suggesting minimal sEMG signal degradation even with consecutive electrode removal and reapplication. (Figure 5b). The largest impedance change for an individual over the four days tested was 11 MΩ at 53 Hz, and 12 MΩ at 151 Hz. The small sample size demonstrates the high inter-subject variability (3.3 MΩ at 53 Hz, and 1.4 MΩ at 151 Hz) in skin impedance between individuals. ICC was 0.62 at 53 Hz (95% confidence interval: 0.0–0.97) and 0.69 at 151 Hz (95% confidence interval: 0.0–0.98), indicating that there was moderate variability between subjects as well as within subjects across the four days.

**Figure 6:**
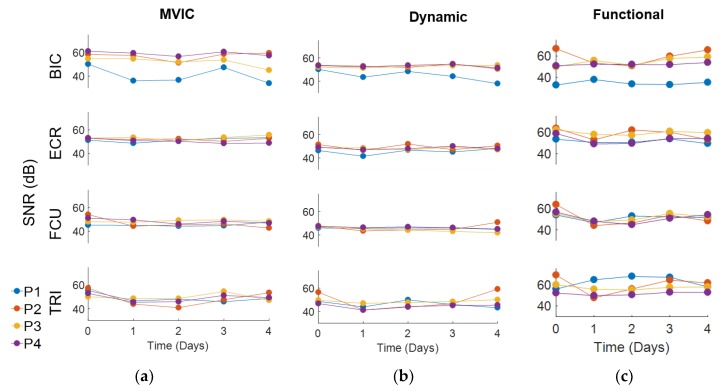
SNR for each muscle group across four days for (**a**) MVIC; (**b**) dynamic and **(c)** functional tests show consistent quality signals for all time points and muscles.

SNR and SMR measurements had no apparent trend over the course of four days for the ESS electrodes (Figure 6) (SNR: 50.5 ± 6.2 dB, SMR: 8.7 ± 8.7 dB). The calculated ICC for SNR was most consistent for BIC (average: 0.73), while other muscle types had a low reliability (ICC < 0.3). However, the ECR, FCU, and TRI had a similar CV to the BIC across the three tests (BIC: 6.7%, ECR: 4.3%, FCU: 5.7%, TRI: 7.6%), suggesting that while intra- and inter- subject variation was comparable, the overall variation was small (less than 10%) of the mean SNR. The minimum SNR measured was 32.9 dB. However, similar to the results obtained in the six-hour test, the average SMR was lower than results obtained from the Delsys electrodes. The minimum and maximum SMR measured was 0.24 dB and 36.5 dB, respectively.

## 4. Discussion

This study demonstrated that ESS electrodes provide high-quality sEMG signals over six hours, comparable to current clinical and research standards for both time and frequency domain measures. Furthermore, there was no significant trend in ESS signal quality over four days of monitoring in a small sample of four subjects. We found that there was moderate variability from day to day within subjects for measures of signal quality, suggesting that further research is required with a larger subject pool to quantify the day-to-day variation of impedance. The linear envelopes for the ESS electrodes were strongly correlated with the linear envelopes of the Delsys electrodes. While the correlation decreased on functional tests for the ECR and FCU as expected due to smaller sEMG amplitudes, the correlations were still high (>0.45) for all muscle types and test conducted over six hours. Importantly, signal correlations did not degrade during the six-hour test, suggesting that ESS electrodes have a comparable signal quality to Delsys electrodes within this time period. 

SNR for the ESS electrode was greater than 25 dB across all tests, and there was no apparent trend in signal quality with an SNR > 30 dB over four days, and the average CV was below 10% for all muscles types and tests conducted. This is much higher than the suggested 15 dB for sEMG data with noise artifacts, and higher than or comparable to SNR reported in previous literature [18,24]. In fact, the average SNR of both the six-hour trial and the four-day trial were close to 50 dB, the SNR of an sEMG signal under ideal conditions [31], and the ESS SNR was significantly higher than the Delsys SNR for the ECR, FCU, and TRI during the six-hour test. In addition, while skin impedance was high, above 100 kΩ at 53 Hz, it was well within the range of literature-reported values for dry electrode impedance [7,10,22,29].

While the implementation of this novel electrode would be most useful for long-term monitoring applications, such as post-stroke rehabilitation, we first chose to evaluate its efficacy with unimpaired subjects. Since the electrodes are not waterproof, the electrodes were tested under real-world conditions, with the electrodes removed and reapplied when subjects showered or exercised. Sweat produced while the electrodes are being worn may decrease skin impedance and influence the performance of the ESS electrodes. However, evaluating the impact of sweat was beyond the scope of this study, and the effect of sweat on ESS performance was reflected in the measurements at each time point. It is worth noting that for the four-day trial, the subject who reported taking the electrodes off the least was also the subject with the most consistent results (P4), whereas the subject who took the electrodes off the most was also the subject with the most variable results (P3). Subjects did not perceive the electrodes to be irritating even after four days of continuous application. The clinical-grade, kind removal silicone tape (3M^TM^) tape, which did not impede subjects’ movement or irritate the skin, provided sufficient skin-electrode contact over four days even for the subjects who reapplied the electrodes twice a day for showering and exercise. The fabrication process allows for the tape to be easily switched out to different types, depending on future applications. Future research could test the electrode when fabricated with athletic tape or waterproof tape.

To inform future clinical translation, we not only quantified the similarity of these sEMG signals for muscle activation measures of sEMG activity such as MVIC and dynamic contraction, but also standard clinical tests used to assess motor impairment after neurologic injuries. These tasks have commonly been excluded from prior research studies evaluating novel electrodes due to lower levels of muscle activity, which poses an additional challenge when evaluating signal quality. However, including these standard clinical tests when evaluating novel electrodes is crucial for the adaptation of these electrodes for clinical and other applications. While average linear envelope amplitude was lower for the functional tests compared to MVICs, SNR and correlations between the Delsys and ESS electrodes were high across six hours. These results suggest that not only can the ESS electrode be used to test standard rehabilitation measures, but also to collect sEMG data for everyday activities which these tests mimic and that the ESS electrode can be easily adapted for use in various scenarios where sEMG data collection is necessary, such as athletics or rehabilitation.

One area of improvement is SMR, which was significantly lower for the ESS electrode compared to the Delsys. While electrodes shifting during the experiment may decrease SMR, we did not observe visible shifting of the ESS electrodes, nor did the subjects report feeling any displaced electrodes during the course of the experiment. Rather, the low SMR was due to the wired interference from the Delsys snap-on leads that was used to simultaneously collect and transmit sEMG signals from the ESS electrodes to the computer. We noticed increased noise in the sEMG data collected when the subject moved and taped down the wires to the subject’s skin using physiological tape to reduce wire movement during testing. However, the connection type can be adjusted based upon the system used for data acquisition, and future development of a wireless data collection unit that interfaces with the ESS electrodes will likely improve SMR while maintaining flexibility and signal integrity. Identifying the best methods for system integration and enabling use with multiple systems is an on-going area of research. In addition, while the ESS electrodes used in this study were fabricated to match the dimensions of the Delsys electrodes for comparison, the size and layout of the electrodes can be easily customized to optimize the electrode for different muscles or applications.

## 5. Conclusions

The results from this study have demonstrated that the ESS electrodes are comparable in performance to conventional dry electrodes commonly used for clinical and research applications such as clinical tests as demonstrated by the Jebsen Taylor Hand Function and Box & Block Tests. Additionally, as evidenced by the four-day trials, their utility is expanded by their capability to provide consistent long-term continuous sEMG data. Subjects were able to wear the ESS electrodes continuously over four days without impeding everyday activities, and independently reapply the electrode multiple times without degradation of signal quality. We confirmed the qualitative similarity in the raw sEMG signal, linear envelope, and power spectral density between the Delsys and the ESS electrodes with the Pearson’s correlation, which averaged 0.89 for all muscles and time points. SNR was high and consistently similar to the Delsys over the course of six hours, but due to wire noise from the Delsys snap on leads, the SMR was lower for the ESS electrode compared to the Delsys. The SNR and SMR for the ESS electrodes averaged 45.1 and 14.4 dB, respectively, across all muscles, time points, and tests conducted. These results suggest that ESS electrodes can provide a low-cost dry electrode that can be worn for extended periods of time without impeding movement. The electrode can be adapted to work with various data acquisition systems and we have shown that the electrode provides quality sEMG measurements tantamount to standard electrodes. Given the similarity in signal measurement between the Delsys and ESS electrodes, as well as the steady signal acquisition over prolonged periods of time, ESS electrodes extend the capacity of dry electrodes to be used for long-term monitoring and evaluation.

## Figures and Tables

**Table 1:** Median correlation values for MVIC, dynamic, and functional tests for each muscle.

	MVIC				Dynamic		Functional	
	0 h	2 h	4 h	6 h	0 h	6 h	0 h	6 h
BIC	0.90	0.93	0.93	0.94	0.90	0.84	0.90	0.91
ECR	0.95	0.96	0.95	0.95	0.88	0.84	0.90	0.85
FCU	0.91	0.93	0.96	0.95	0.91	0.91	0.87	0.87
TRI	0.91	0.87	0.89	0.87	0.84	0.83	0.82	0.81
